# Controlled Lateral Pressure on Cortical Bone Using Blade-Equipped Implants: An Experimental Study in Rabbits

**DOI:** 10.3390/bioengineering11080835

**Published:** 2024-08-16

**Authors:** Vitor Ferreira Balan, Mauro Ferri, Eduardo Pires Godoy, Leticia Gabriela Artioli, Daniele Botticelli, Erick Ricardo Silva, Samuel Porfirio Xavier

**Affiliations:** 1 Department of Oral and Maxillofacial Surgery and Periodontology, Faculty of Dentistry of Ribeirão Preto, University of São Paulo, Av. do Café-Subsetor Oeste-11 (N-11), Ribeirão Preto 14040-904, SP, Brazil; vitor.balan@usp.br (V.F.B.); leticia.artioli@usp.br (L.G.A.); erick.silva@usp.br (E.R.S.); spx@forp.usp.br (S.P.X.); 2 Private Practice, Cartagena de Indias 130001, Colombia; medicina2000ctg@hotmail.com; 3 Department of Basic and Oral Biology, Ribeirão Preto School of Dentistry, University of São Paulo, Ribeirão Preto 14040-904, SP, Brazil; eduardo.godoy@usp.br; 4 ARDEC Academy, 47923 Rimini, Italy

**Keywords:** animal study, bone healing, histology, cortical layer, marginal gap

## Abstract

Background: This study aimed to evaluate the biological behavior of a novel implant design incorporating decompressive cervical blades. Hence, the aim of the present study was to evaluate the healing outcomes in cortical regions where decompressive protocols were implemented using implants equipped with blades and installed applying a bicortical anchorage. Materials and Methods: Blades with varying diameters were integrated into the coronal portion of the implant to prepare the cortical region of rabbit tibiae. The blade diameters differed from the implant collar by the following amounts: control group (0 µm), +50 µm, and +200 µm. Results: No marginal bone loss was detected. Instead, all implants exhibited new bone formation in the coronal region. Complete closure was observed in the CG-0 group, as well as in the TG-50 and TG-200 groups, despite the presence of marginal gaps without primary bone contact at installation. In the apical region, most implants breached the cortical layer. Nevertheless, new bone formation in this region completely closed the osteotomy, effectively isolating the internal environment of the tibia from the external. Conclusions: The use of a blade incorporated into the implant body enabled precise preparation of the cortical layer, allowing for controlled decompression in the targeted area. This technique resulted in optimal osseointegration with no loss of marginal bone, and complete restoration of marginal gaps ranging from 0 µm to 200 µm.

## 1. Introduction

Osseointegrated implants are extensively employed in esthetic and functional restorations for individuals affected by partial or total edentulism. Recent research indicates that titanium possesses properties that promote interaction with the surrounding bone tissue [1,2], giving implants mechanical characteristics suitable for withstanding the demands of masticatory loads and physiological stimuli during the chewing process [3].

Despite the significant success rates documented with implants, a variety of studies have been conducted with the aim of optimizing osseointegration through the application of different technologies and manufacturing methods. These innovations focus on modifying the surface microtopography [4,5] through physicochemical means and the design of implants with respect to macrogeometry [6,7,8]. Preclinical studies demonstrate that these alterations significantly improve the biological performance of implants [9,10].

Bone compression during the installation of integrated bone implants plays a fundamental role in the primary stability of the implant [11]; however, excessive compressions can induce distortions in the peri-implant bone, resulting in adverse effects on the local microcirculation and increasing the risk of bone necrosis, leading to implant failure [12,13]. Additionally, compressive insertions may also be associated with pain and resorption of the bone crest adjacent to the implants [14,15,16].

Recent research suggests that reduced levels of bone compression around the implant are associated with increased osseointegration [17,18]. Therefore, it is crucial that an ideal implant is designed to maintain an appropriate balance between tensile and compressive forces [19]. Furthermore, it has been observed that high insertion torques can lead to increased bone compression around implants, resulting in significant marginal bone resorption and subsequent implant failure [20].

It was further shown that modifying the macrogeometry of implants by incorporating a decompression chamber in the threads resulted in an improvement in the osseointegration process [21,22].

For experimental investigations of the interaction between implants and bone tissue, as well as to assess the impact of implant macrogeometry on new bone formation, the animal model using rabbit tibia has been widely utilized [23]. The tibia of these animals presents notable distinctions in topographic and morphological aspects, as it can be divided into two well-defined regions: the diaphysis, characterized by the presence of cortical bone and a medullary space, resembling type II bone; and the metaphysis, composed of trabecular tissue resembling type III bone. The variation in bone morphology and density between these two regions is related to the amount of bone formation occurring in each of them [24,25]. In an experimental study on canines [26], implants analogous to those utilized in the present investigation were placed in the healed alveolar ridge of the mandible. These implants featured blades positioned coronally, just below the collar, designed to induce standardized compression and decompression on the cortical bone layer. The study observed that higher levels of osseointegration occurred when there was no or minimal gap around the implant in the coronal region. Despite the presence of marginal gaps, implant stability was maintained due to the thickness of the cortical bone layer and the density of the spongiosa. However, in scenarios involving a thin cortical layer and low bone density in the marrow compartments, that might be mimicked by the rabbit tibiae, stability may be compromised. Under these conditions, bicortical implant insertion can address the issue.

Therefore, the aim of the present study was to evaluate the healing outcomes in cortical regions where decompressive protocols were implemented using implants equipped with blades and installed applying a bicortical anchorage.

## 2. Materials and Methods

### 2.1. Ethical Statements

The protocol for this study was approved on the 23 August 2022 by the Ethics Committee of the Ribeirão Preto Dental School at the University of São Paulo—CEUA (protocol # 2022.1.534.58.0). Brazilian guidelines for animal experiments were adhered to. The study was conducted in accordance with ARRIVE guidelines.

### 2.2. Study Design

A total of 48 specially manufactured titanium implants with a novel design of different decompressive cervical blade profiles were used for this research. The implants received conventional surface treatment recommended by the company, involving a double acid-etching process, and have a surface roughness of Ra = 1.3 μm (Leader Medica—Medical Technology—Pádua, Italy). They have a diameter of 3.75 mm and a height of 10 mm, divided into three groups based on differences in the size of the decompressive cervical blades:

Control Group (CG-0): Neutral blade (0.0 mm)/no radial difference;
Test Group 1 (TG-50): Blades with a radial difference of +0.05 mm, resulting in mild bone decompression;
Test Group 2 (TG-200): Blades with a radial difference of +0.2 mm, resulting in greater bone decompression.

**Figure 1:**
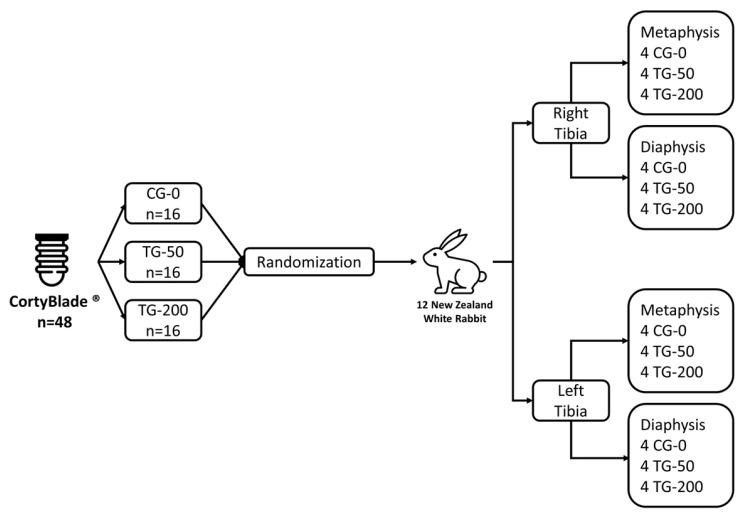
Schematic representation of the study design. Each animal received four implants: one of each type plus one randomly selected from the three groups.

Two implants were installed in the medial segment of the tibia bilaterally, with distribution and randomization into the three different experimental groups, in the metaphyseal and diaphyseal regions of each animal, following the methodology described by Caneva et al., 2014 [24] (Figure 1). The osteotomies were performed uniformly in all experimental sites, following the sequence of drills recommended by the manufacturer: Starter, 2.40 mm; 2.80 mm; 3.2 mm; 3.4 mm; 3.65 mm; 3.75 mm (Leader Medica—Medical Technology—Pádua, Italy).

### 2.3. Experimental Animals and Sample Size

An experimental, prospective, and randomized study was conducted with the test and control implants in the same animal, eliminating interference between individuals within the same group and allowing for the use of a reduced number of animals with sample representativeness.

A previous study in dogs evaluated the influence of different torques (70 Ncm vs. 30 Ncm) on implant osseointegration, in which higher torque meant more compression on the recipient bone [18]. A mean difference in bone-to-implant contact of 9.4% was observed in favor of the lower torque. With a calculated effect size of 2.57, applying an α = 0.05, and a power of 0.9, a two-tails evaluation resulted in a sample size of 5 pairs of animals to reject the null hypothesis that the difference is zero (G*Power 3.1.9.4). However, considering multiple comparison corrections and possible complications and death of animals, the number was increased to 12 animals. Hence, 12 adult female New Zealand White rabbits weighing 3.5–4.0 kg and approximately 6 months old were used.

### 2.4. Randomization and Allocation Concealment

Randomization of the test and control groups were conducted electronically by an author not involved in the selection and handling of animals and/or surgical procedures (S.P.X.). Treatment allocations were secured in sealed opaque envelopes and were revealed to the surgeon (not involved in the study randomization) only at the time of implant placement.

### 2.5. Implant Characteristics

**Figure 2:**
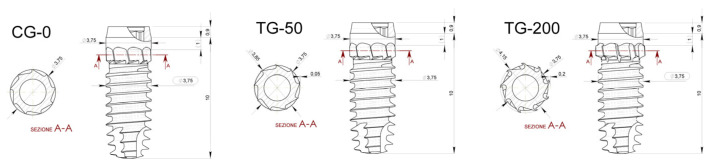
Technical design of the osseointegrated implants (CortyBlade^®^ Leader Medica, Italy). GC-0—Implant with neutral blade (without radial difference). TG-50—Implant with decompressive blades with a radial difference of +50 μm. TG-200—Implant with decompressive blades with a radial difference of +200 μm.

The implants used (CortyBlade^®^ Leader Medica s.r.l., via Giacinto Andrea Longhin 11, 35129 Padova, PD, Italy) were 3.75 mm in diameter and 8.5 mm in length with a tapered one-piece conformation with a double acid-etched surface. The transmucosal collar was 1.8 mm high, with a convergent conformation. The CortyBlade implants were equipped with blades in the coronal aspect aiming to create bone decompression in the cortical layer. Implants with four different blade diameters were used. The difference in the diameter of the blades in relation to the neck of the implant was as follows: 0 μm (control site; CG-0), +50 μm (test site; TG-50), or +200 μm (test site; TG-200) (Figure 2).

### 2.6. Anesthesia and Medication Procedures

Sedation was performed with Acepromazine 1.0 mg/kg (Acepran^®^, Vetnil, Louveira, Brazil) administered intramuscularly (IM). Subsequently, anesthesia was induced with Xylazine 3.0 mg/kg (Dopaser^®^, Hertape Calier, Juatuba, Brazil) and Ketamine Hydrochloride 50 mg/kg (Ketamin Agener, União Química Farmacêutica Nacional S/A, Embu-Guaçu, Brazil) IM. Local anesthesia was performed using 2% Mepivacaine with 1:100,000 Epinephrine (Mepiadre, Nova DFL, Rio de Janeiro, Brazil) in the experimental regions. In the preoperative period, animals received a prophylactic dose of Oxytetracycline 20 mg/kg IM (Biovet, Vargem Grande Paulista, Brazil), 0.2% Meloxicam (Framavet, 1.0 mg/kg, s.c.; União Química Farmacêutica Nacional S/A., Embu-Guaçu, Brazil), and Tramadol Hydrochloride 5.0 mg/kg s.c. (Halexlstar; Goiânia, Brazil). Anti-inflammatory (Meloxicam 0.2%, Flamavet, 0.5 mg/kg, s.c.; União Química Farmacêutica Nacional S/A., Embu-Guaçu, Brazil) and analgesic medications (Tramadol Hydrochloride, 5.0 mg/kg, s.c., Halex Istar, Goiânia, Brazil) were administered once daily for the first two postoperative days.

### 2.7. Surgical Procedure

The areas to be operated were shaved, and antiseptic preparation was performed by topically applying 1% Povidone-Iodine solution (Riodeine Tincture, Rioquímica, São José do Rio Preto, Brazil). Local anesthesia was administered as described above. All surgeries were performed by a single experienced and trained operator.

**Figure 3:**
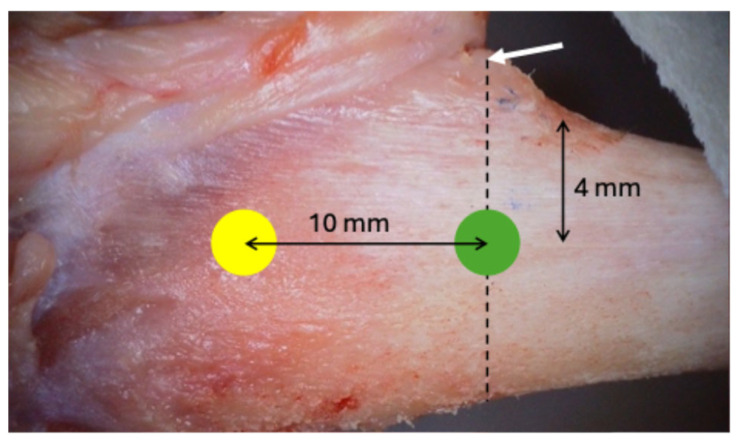
Anatomical specimen demonstrating reference regions for selecting experimental sites. The white arrow indicates the tibial tuberosity that serves as a reference for the tibial diaphysis region. The green circle represents the selection of the experimental site in the tibial diaphysis. The yellow circle is 10 mm away from the green circle, indicating the experimental site in the tibial metaphysis.

A linear incision of 2.5 to 3 cm was made on the skin in the medial segment of the tibia bilaterally, and the skin and periosteum were retracted. Two experimental sites were identified in each tibia, the metaphyseal and diaphyseal regions, approximately 10 mm apart (Figure 3).

**Figure 4:**
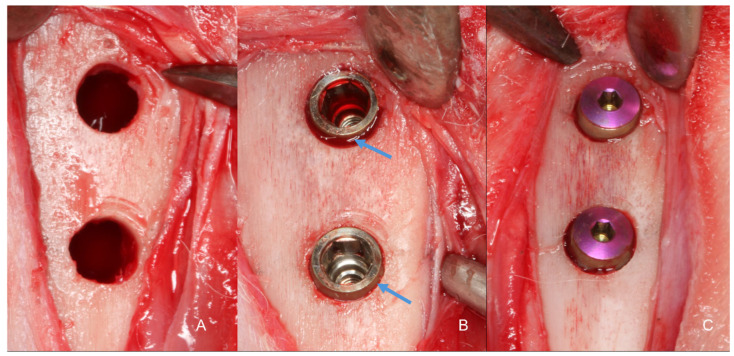
Intraoperative images. (**A**): Surgical site prepared for implant installation (below the diaphysis region and above the metaphysis region). (**B**): Implants installed in their final position. The blue arrow shows the region decompressed by the Cortyblade. (**C**): Cover screws installed on the implants.

Identical osteotomies using the predetermined drill sequence are shown in Figure 4A. At this point, the treatment allocation was revealed to the surgeon. Implants with modified blade designs were inserted so that their prosthetic platform was positioned at about the bone level (Figure 4B), and their apices were anchored in the inner cortex of the tibia, following the protocol described by Caneva et al., 2015 [25]. Torque insertion measurements at the final position were recorded using a wrench included in the surgical kit. Cover screws were placed (Figure 4C) and sutures were provided in layers using 4-0 Nylon (Ethicon^®^, Johnson & Johnson^®^, São José dos Campos, São Paulo, Brazil), and a bandage strip was placed over the wound, remaining in place for three days of the postoperative period.

### 2.8. Animal Maintenance

For a preoperative adaptation period of two weeks and throughout the postoperative period, the rabbits were kept in individual cages without specific bedding, but with a small plastic support for resting their paws (1 animal/6000 cm^2^). The room where they were housed had a split air conditioner (21 °C) without humidity control, and 27 to 34 air changes per hour were performed. There was an automatic lighting control every 12/12 h, and ad libitum filtered food and water were provided. Sanitary barriers, including an autoclave, sanitary facilities/locker rooms, and insect control screens, were available. After the surgical procedure, the rabbits were placed in specific cages for motion control for two days, and then they returned to their cages, being observed daily for signs of pain and/or infection at the surgical wound site until the time of euthanasia.

### 2.9. Euthanasia

After 10 weeks of the postoperative period, euthanasia was performed (*n* = 12). Firstly, sedation was administered with Acepromazine 1.0 mg/kg (Acepran^®^, Vetnil, Louveira, São Paulo, Brazil), followed by anesthesia through the combination of Xylazine 3.0 mg/kg (Dopaser^®^, Hertape Calier, Juatuba, Minas Gerais, Brazil) and Ketamine Hydrochloride 50 mg/kg (Ketamin Agener, União Química Farmacêutica Nacional S/A., Embu-Guaçu, São Paulo, Brazil), administered IM. Subsequently, the animals were individually placed in a CO^2^ chamber with controlled flow of 7 L/min at a rate of 20% of the total volume. This flow was maintained for at least 1 min after confirming clinical death of the animal, checking for signs of respiratory arrest, mucosal cyanosis, and absence of a pulse. Biopsies of the operated sites were collected and immediately immersed in 10% paraformaldehyde solution.

### 2.10. Histological Processing

The specimens were dehydrated in a sequence of alcohol solutions and then embedded in resin (LR WhiteTM HardGrid, London Resin Co., Ltd., Berkshire, UK). After polymerization, each block was cut in a coronal plane guided by the center of the implant. Two sections of approximately 100–150 μm were prepared using a precision cutting device (Exakt, Apparatebau, Norderstedt, Germany) and sanded until approximately 50–60 μm thick sections were obtained. Histological sections were stained using Toluidine Blue, Stevenel’s Blue, and Alizarin Red.

### 2.11. Histological Evaluation

**Figure 5:**
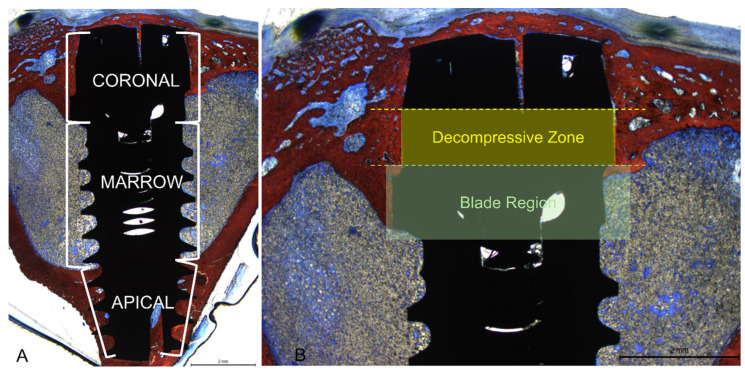
(**A**): Photomicrograph demonstrating the total length of the implant and the three regions evaluated: coronal, marrow, and apical. (**B**): Photomicrograph under 16× magnification of the implant coronal region. In yellow, the decompressive zone in the cervical collar is represented. In green, the region of the cortical blades.

All measurements were made by a single examiner (V.B.F.) not involved in the surgical procedure and without knowledge of the previous randomization of the study. Before starting the histological measurements, a specialist (S.P.X.) calibrated the examiner responsible for the analyses until obtaining an inter-examiner kappa > 0.9. Images were taken at about ×100 magnification. Histological evaluations were conducted in three implant regions (coronal, marrow, and apical; Figure 5A) using Image J software version 1.54d (NIH, Bethesda, MD, USA). In the coronal region, bone-to-implant contact was measured in two different regions, i.e., the collar region (decompressive zone) and the blade region (Figure 5B).

Mean values were obtained to express the BIC% in the coronal region. New bone was also evaluated in the apical region between the coronal and apical extensions of integration. Osseointegration was also evaluated in the marrow compartment from the base of the blades and the internal limits of the apical cortical layer. Newly formed bone and soft tissues (medullary spaces and osteons) were evaluated.

### 2.12. Statistical Analysis

Four implants were inserted into each animal tibia, with one group out of three randomly receiving two implants per animal. A mean value of these two implants was calculated so that each group was represented once for each animal (*n* = 12). The primary variable was the percentage of bone in contact with the implant surface in the decompressive region, while osseointegration extension in the coronal region and BIC% in other regions were considered secondary variables. Data normality was evaluated using the Shapiro–Wilk test. Depending on the results, either an ANOVA or a Friedman test was applied for group comparisons. Differences were analyzed using a paired t-test or a Wilcoxon test. Data were stored in an Excel file (Microsoft^®^ Excel^®^ V. 2404) for descriptive statistics. Statistical analyses were conducted using Prism Software v. 10 (GraphPad Software, LLC, San Diego, CA, USA), with an alpha level of 5%.

## 3. Results

### 3.1. Clinical Outcomes

All animals healed uneventfully, and all implants were available for histological processing. No implants were lost resulting in *n* = 12.

### 3.2. Descriptive Histological Evaluation

**Figure 6:**
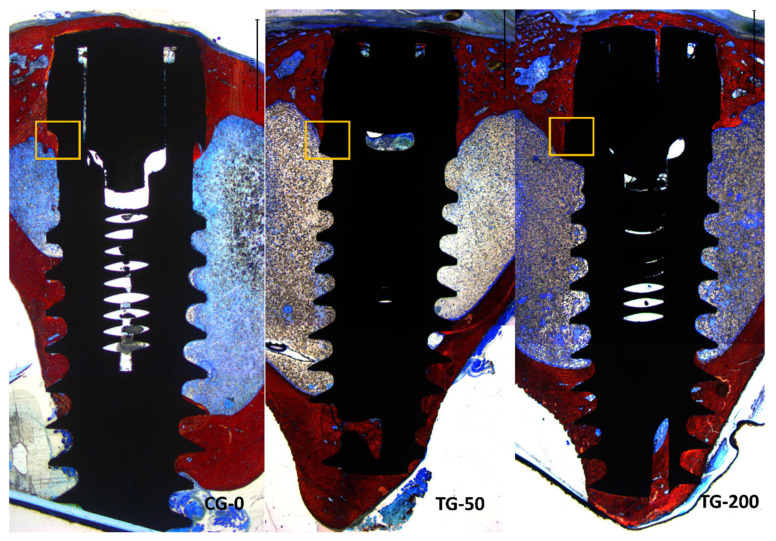
Photomicrograph of ground sections showing healing at the three different implants. CG-0 represents the implant with the blades without radial difference. TG-50 represents the implant with blades with a radial difference of +50 μm. TG-200 represents the implant with blades with a radial difference of +200 μm. The orange squares indicate the sites where the blades are located.

All histological slides were available for analysis. All implants presented optimal osseointegration without marginal bone loss (Figure 6).

**Figure 7:**
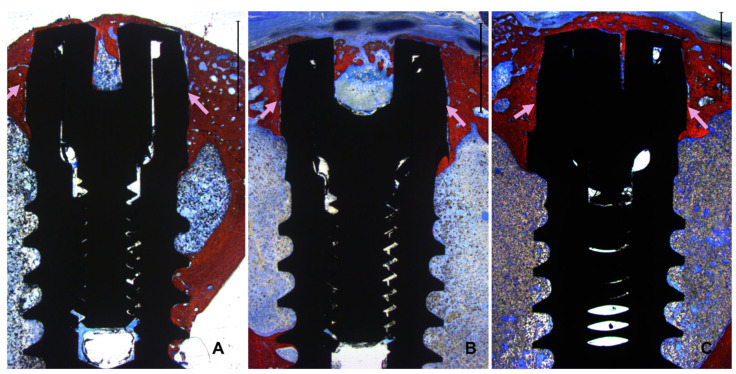
Photomicrograph of ground sections showing healing at the three different implants. (**A**): CG-0; (**B**): TG-50; (**C**): TG-200. The pink arrows illustrate the decompressed region completely filled with new bone.

Instead, all implants exhibited new bone formation in the coronal region. Complete closure was observed in the CG-0 group (Figure 7A), as well as in the TG-50 (Figure 7B) and TG-200 (Figure 7C) groups, despite the presence of marginal gaps without primary bone contact at installation.

**Figure 8:**
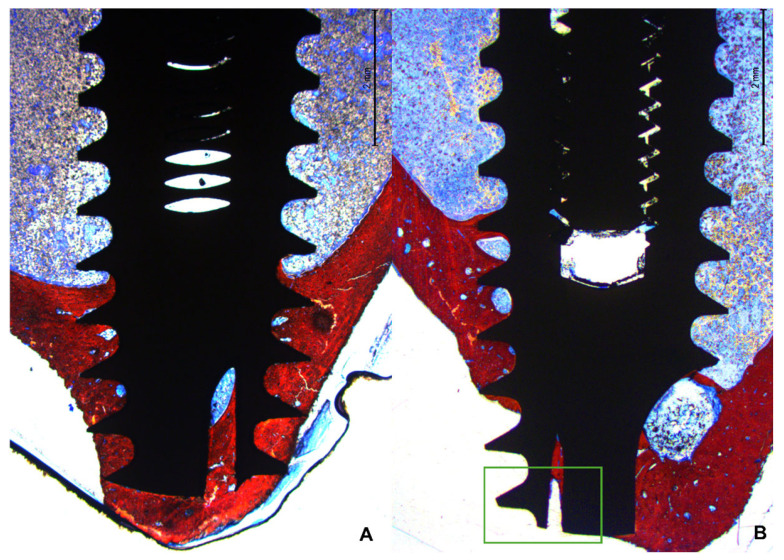
Photomicrograph under 16× magnification of the implant apical region. (**A**): Implant apical area with apex perfectly inserted at the cortical layer. (**B**): Apical region of the implant crossing the cortical layer. A complete closure of the region with new bone is observed. The green rectangle indicates the apical portion of the implant beyond the cortical bone layer of the tibia.

In the apical region, several implants breached the cortical layer (Figure 8A,B). Nevertheless, new bone formation in this region completely closed the osteotomy, effectively isolating the internal environment of the tibia from the external.

**Figure 9:**
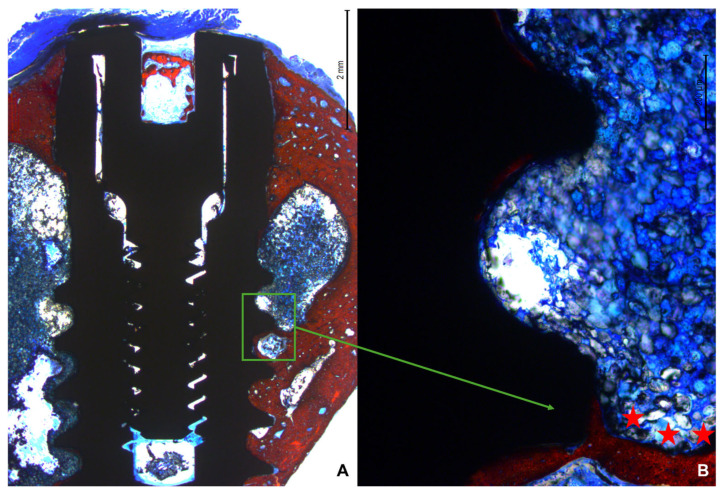
(**A**): Photomicrograph under 16× magnification. (**B**): Photomicrograph under 100× magnification. The region highlighted in green demonstrates bone formation close to the implant thread. The ★ symbols indicate the bone forming from the lateral bone cortex.

The newly formed bone predominantly accumulated near the two cortical layers, spreading toward the marrow region, which generally lacked new bone between the coronal and apical regions. Only when the implant was close to the lateral cortical walls did new bone extend into the marrow region between the cortex and the implant surface (Figure 9A,B).

No significant differences in the healing process were observed between implants placed in the diaphysis and those near the metaphysis.

### 3.3. Histomorphometric Assessments

In the decompressive region (collar), similar amounts of newly formed bone were observed in all groups, with the mean values ranging between 60.6 and 63.6% (Table 1). In the blade zone, statistically significant higher proportions of new bone were found in the CG-0 group (53.4%) compared to TG-50 (33.6%) and TG-200 (34.4%). Merging the data between the decompressive and blade zone, no statistically significant differences were obtained.

The mean value of the cortical layer was 1.31 ± 0.29 mm. The apical extension of osteointegration from the implant shoulder considering all implants was 2.68 ± 0.33 mm for CG-0, 2.41 ± 0.27 mm for TG-50, and 2.32 ± 0.38 mm for TG-200.

High fractions of new bone were found also in the apical region in all implants, with the mean values ranging between 71.8 and 78.8%. Higher percentages were found at the implant with marginal defects.

In the marrow space, very low percentages of new bone were found in all groups, with the mean value ranging between 9.4 and 10.4%. The total percentage of new bone on the implant surface was similar in all groups, with the mean values ranging between 38.6 and 42.0%.

## 4. Discussion

The decompression zone exhibited a similar amount of osseointegration across all groups, even those with marginal defects. However, in the blade region, the CG-0 group demonstrated higher osseointegration compared to the test groups. Additionally, a high percentage of osseointegration was observed in the apical region across all groups.

The use of blades in the cortical region was meant to reduce the compression at the cortical layer. The compression of this region might create strain in the peri-implant bone, negatively impacting osteocyte survival. This high strain restricts blood flow and results in microdamage to the bone, leading to osteocyte necrosis, significant bone remodeling, and limited new bone formation [27]. Blades of three different dimensions were utilized to widen the coronal aspect of the osteotomies in the decompression region, matching the diameter of the collar or leaving a residual gap of either 50 µm or 200 µm after implant installation. The cortical regions in all specimens, including those with an initial gap, were completely filled with newly formed bone. The implants were positioned so that the prosthetic platform was close to the cortical layer, with the intention of having the blades pass beyond the lower limit of the cortical layer to prevent contact between the two structures. Specifically, the coronal level of the blade was fabricated 1.9 mm from the prosthetic platform, while the mean width of the cortical layer was 1.3 mm. Despite the absence of primary contact between the collar and the cortical layer, it can be assumed that the gap between the collar and the osteotomy in the two test groups was filled with new bone through distance or contact osteogenesis [28,29]. This result is in agreement with a study in which a chamber 0.4 mm in depth was created around implants with either a moderately rough or a turned surface [21,30]. New bone was found on the implant surface within the chamber already after 1 week. A higher percentage of new bone formation was observed on the rough surface compared to the turned surface. In another similar experiment, chambers deeper than 0.5 mm were prepared around the implants [22]. Again, new bone was found on the implant surface after 2 weeks of healing. Although the distances in the aforementioned studies were significantly greater than those used in the present study, a key difference is that no contact between the bone and implant surface was permitted in the present study, whereas in the chamber studies, the threads near the chambers were in close contact with the bone walls of the osteotomy. New bone formation could have occurred from the osteotomy and spread onto the implant surface due to the osteoconductive properties of the implant surface. Indeed, in other experiments where marginal circumferential defects of 0.5–1.25 mm were created around implants, it was demonstrated that new bone formation occurred from the lateral walls [31,32], reaching a distance of 0.4 mm from the implant surface within 20 days, regardless of the initial size of the gap [33]. The remaining 0.4 mm defect was subsequently closed over time by new bone forming on the implant surface from the bottom of the defect where the implant was in close contact with the bone. This process was facilitated by the osteoconductive properties of the implant surface [34]. In contrast, other experimental studies [35,36] observed minimal integration when no contacts were allowed between the bone walls of the osteotomy and the body of the implant, especially in defects ranging from 0.7 to 1.20 mm or 0.35 to 0.85 mm. The formation of woven bone was halted at around 0.4–0.5 mm from the implant surface, preventing direct contact with the implant surface [35]. However, in the present study, complete closure of the gap around the collar of the implant was achieved, even in the absence of primary bone contact. This suggests that new bone formation, either through distance or contact osteogenesis, occurred and integrated onto the implant surface.

Indeed, a notable distinction in the present study compared to the previously mentioned ones is the smaller dimension of the gaps, which were less than 0.4 mm (50 µm and 200 µm). This short “jumping distance”, as described by Botticelli [32], may facilitate bone formation on the implant surface through contact or distance osteogenesis [28,29]. Another unique aspect is the formation of bone chips by the blades, which fill the gap around the implant collar, as observed in a dog experiment [26]. It has been demonstrated that bone debris can serve as bridges for the formation of osteoid tissue and newly formed bone, as supported by human [37,38] and animal studies [39]. These factors likely contributed to the observed osseointegration and closure of the gaps around the implant collar in the present study.

It is worth noting that implants with blades have previously shown success in experiments conducted in dogs in which different compression or decompression effects were produced in the cortical layer [26]. In that experiment, in all groups, the cortical region where the blades executed their cutting action exhibited regular healing, achieving optimal hard and soft tissue sealing. The lowest marginal bone resorption was observed at the 50 µm marginal gap while the best osseointegration was obtained in both the 50 µm and 200 µm marginal gaps. Bone particles accumulated around the cortical blades, especially in the +200 μm group, and were integrated into the newly formed bone. These findings from the current experiment endorse the use of blades that create a marginal gap of 50 μm upon implant insertion.

The greater extension of osseointegration in an apical direction observed in the control group compared to the test groups, along with the higher percentage of bone-to-implant contact (BIC%), suggests potentially favorable outcomes with the CG-0 group. Additionally, the similar BIC% in the decompression zone further supports this notion.

The present study demonstrated that all implants were successfully integrated in both cortical layers, with higher bone-to-implant contact percentages (BIC%) observed in the apical regions compared to the coronal regions. This apical integration suggests optimal initial implant stability, even in the absence of contact in the coronal region, in both the TG-50 and TG-200 groups. This finding aligns with observations from other experiments [24,25,40] and provides further support to the outcomes reported in clinical studies where bicortical installation was employed [41,42].

The primary limitations of the present study pertain to the phylogenetic differences between rabbits and humans, as well as the distinct anatomical and physiological characteristics of the tibia compared to the alveolar bone crest. Consequently, any inferences should be confined to the histological findings and not directly extrapolated to clinical settings. Nonetheless, the data generated from this study provide a valuable foundation for subsequent clinical research aimed at confirming these findings.

## 5. Conclusions

The use of a blade incorporated to the implant body enabled precise preparation of the cortical layer, allowing for controlled decompression in the targeted area. This technique resulted in optimal osseointegration with no loss of marginal bone, and complete restoration of marginal gaps ranging from 0 µm to 200 µm.

This is the second animal study that showed optimal healing of the calibrated decompressive gaps produced at the cortical layer using implants equipped with cortical blades. This collective evidence underscores the potential efficacy and versatility of this type of implant in promoting osseointegration and minimizing marginal resorption. Further complementary data on bone remodeling in humans and clinical long-term results are necessary to support the findings from the present study.

## Figures and Tables

**Table 1:** Bone-to-implant contact percentage in the various region analyzed. Mean values ± standard deviation. * Statistical difference between the test and control groups (TG vs. GC) = *p* < 0.05.

Groups	Coronal	Decompressive	Blades	Marrow	Apical	Total
CG-0	57.0 ± 15.0	60.6 ± 16.5	53.4 ± 23.3 *	9.4 ± 4.4	71.8 ± 12.8	38.6 ± 7.3
TG-50	48.6 ± 10.3	63.6 ± 18.0	33.6 ± 14.7 *	10.0 ± 6.2	77.8 ± 11.4	40.7 ± 6.5
TG-200	47.8 ± 12.5	61.2 ± 16.2	34.4 ± 15.5 *	10.4 ± 11.5	78.8 ± 7.2	42.0 ± 8.3

## Data Availability

Data are available on reasonable request.
